# Supplementary material for: Multi-Institutional Evaluation of Pathologists’ Assessment Compared to Immunoscore
Source: Cancers (Basel). 2023 Aug 10;15(16):4045. doi: 10.3390/cancers15164045 (PMC10452341; doi:10.3390/cancers15164045)
Supplement: Supplementary file 1 [file cancers-15-04045-s001.zip › cancers-2506162-supplementary.pdf]

Cohort Characteristics table			
Age			
	N	270	(100%)
	Mean (SD)	69.7	(13.3)
	Median (Q1-Q3)	72	(61 - 79.8)
	Range (min-max) (95CI)	25 - 95	(68.1 - 71.3)
Gender			
	1-Male	122	(45.2%)
	2-Female	148	(54.8%)
T.STAGE			
	T1	13	(4.8%)
	T2	30	(11.1%)
	T3	183	(67.8%)
	T4	44	(16.3%)
N.Stage			
	N0	197	(73%)
	N1	50	(18.5%)
	N2	18	(6.7%)
	Not Available	5	(1.9%)
TOTAL.LN			
	N	270	(100%)
	Mean (SD)	13.6	(10.8)
	Median (Q1-Q3)	10	(6 - 18)
	Range (min-max) (95CI)	0 - 62	(12.3 - 14.9)
POS.LN			
	N	68	(100%)
	Mean (SD)	3.3	(4.5)
	Median (Q1-Q3)	2	(1 - 4)
	Range (min-max) (95CI)	1 - 30	(2.2 - 4.4)
UICC.STAGE			
	I	37	(13.7%)
	II	160	(59.3%)
	III	68	(25.2%)
	Not Available	5	(1.9%)
ChemoPostop			
	NO	193	(71.5%)
	YES	77	(28.5%)
HighRisk-T4-Or-VELIPI			
	HighRisk-T4-Or-VELIPI	92	(34.1%)
	LowRisk	173	(64.1%)
	NaN	5	(1.9%)
VELIPI			
	1-NO	205	(75.9%)
	2-YES	60	(22.2%)
	Not Available	5	(1.9%)
Differentiation			
	1-well	180	(66.7%)
	2-moderate	75	(27.8%)
	3-poor-undiff	12	(4.4%)
	Not Available	3	(1.1%)
Mucinous.colloide			
	1-NO	198	(73.3%)
	2-YES	66	(24.4%)
	Not Available	6	(2.2%)
Sidedness			
	1-proximal	124	(45.9%)
	2-distal	144	(53.3%)
	Not Available	2	(0.7%)
CD3CT-mean-QC			
	N	270	(100%)
	Mean (SD)	561.6	(398.4)
	Median (Q1-Q3)	453.9	(271.8 - 741.4)
	Range (min-max) (95CI)	5.6 - 2398.8	(514.1 - 609.1)
CD3IM-mean-QC			
	N	270	(100%)
	Mean (SD)	950.3	(625.2)
	Median (Q1-Q3)	791.4	(470.6 - 1283.3)
	Range (min-max) (95CI)	15.5 - 4142.3	(875.8 - 1024.9)
CD8CT-mean-QC			
	N	270	(100%)
	Mean (SD)	173.9	(200.5)
	Median (Q1-Q3)	113.1	(59.7 - 219.4)
	Range (min-max) (95CI)	6.7 - 1712	(150 - 197.8)
CD8IM-mean-QC			
	N	270	(100%)
	Mean (SD)	324.6	(313.2)
	Median (Q1-Q3)	236.1	(121.1 - 424.8)
	Range (min-max) (95CI)	12.1 - 2228.6	(287.3 - 362)
IS-2Level-TS-cut			
	0-25%	90	(33.3%)
	25-100%	180	(66.7%)
IS-3Level-TS-cut			
	0-25%	90	(33.3%)
	25-70%	131	(48.5%)
	70-100%	49	(18.1%)
IS-5Level-TS-cut			
	0-10%	33	(12.2%)
	10-25%	57	(21.1%)
	25-70%	131	(48.5%)
	70-95%	46	(17%)
	95-100%	3	(1.1%)

Table S1. Demographic distribution.

Table S2. Comparison of Immunoscore Unsupervised classification of Pathologists *vs* Digital Immunoscore

Lo *vs* Int *vs* Hi* / Classification**

T Stage

	T 1-2 (43pts)		T 3 (183pts)		T 4 (44pts)		T 3-4 (227pts)	
**Pathologist**	**Kappa**	**Concordance**	**Kappa**	**Concordance**	**Kappa**	**Concordance**	**Kappa**	**Concordance**
1	0.165	none	0.478	weak	0.148	none	0.424	weak
2	0.333	minimal	0.497	weak	0.420	weak	0.488	weak
3	0.335	minimal	0.411	weak	0.341	minimal	0.402	weak
4	0.394	minimal	0.392	minimal	0.305	minimal	0.378	minimal
5	0.119	none	0.420	weak	0.494	weak	0.436	weak
6	0.302	minimal	0.497	weak	0.374	minimal	0.478	weak
7	0.173	none	0.446	weak	0.324	minimal	0.428	weak
8	0.364	minimal	0.432	weak	0.365	minimal	0.422	weak
9	0.548	weak	0.533	weak	0.397	minimal	0.513	weak
10	0.284	minimal	0.268	minimal	0.169	none	0.258	minimal
**Mean**	**0.302**	**minimal**	**0.438**	**weak**	**0.334**	**minimal**	**0.423**	**weak**

Mucinous Colloid Type

	Yes (66pts)		No (198pts)	
**Pathologist**	**Kappa**	**Concordance**	**Kappa**	**Concordance**
1	0.269	minimal	0.412	weak
2	0.482	weak	0.457	weak
3	0.398	minimal	0.385	minimal
4	0.370	minimal	0.391	minimal
5	0.450	weak	0.359	minimal
6	0.478	weak	0.457	weak
7	0.407	weak	0.382	minimal
8	0.351	minimal	0.442	weak
9	0.594	weak	0.495	weak
10	0.295	minimal	0.259	minimal
**Mean**	**0.409**	**weak**	**0.404**	**weak**

Tumor Differentiation

	Well (180pts)		Moderate (75pts)		Poor/Undiff (12pts)	
**Pathologist**	**Kappa**	**Concordance**	**Kappa**	**Concordance**	**Kappa**	**Concordance**
1	0.368	minimal	0.339	minimal	0.861	strong
2	0.468	weak	0.457	weak	0.582	weak
3	0.385	minimal	0.348	minimal	0.711	moderate
4	0.374	minimal	0.367	minimal	0.488	weak
5	0.379	minimal	0.351	minimal	0.617	moderate
6	0.441	weak	0.454	weak	0.604	moderate
7	0.344	minimal	0.444	weak	0.609	moderate
8	0.474	weak	0.329	minimal	0.368	minimal
9	0.538	weak	0.491	weak	0.593	weak
10	0.221	minimal	0.399	minimal	0.250	minimal
**Mean**	**0.399**	**minimal**	**0.398**	**minimal**	**0.568**	**weak**

*** Each comparison is done for the pathologist's classification vs. the Gold-Standard Immunoscore for the same sample and measured by Cohen's Kappa. ** Kappa: worse than random (negative Kappa), none (0-0.2), minimal (0.21-0.39), weak (0.4-0.59), moderate (0.6-0.79), strong (0.8-0.9), and almost perfect (>0.9). Mean: mean Kappa score accross 10 pathologists.**

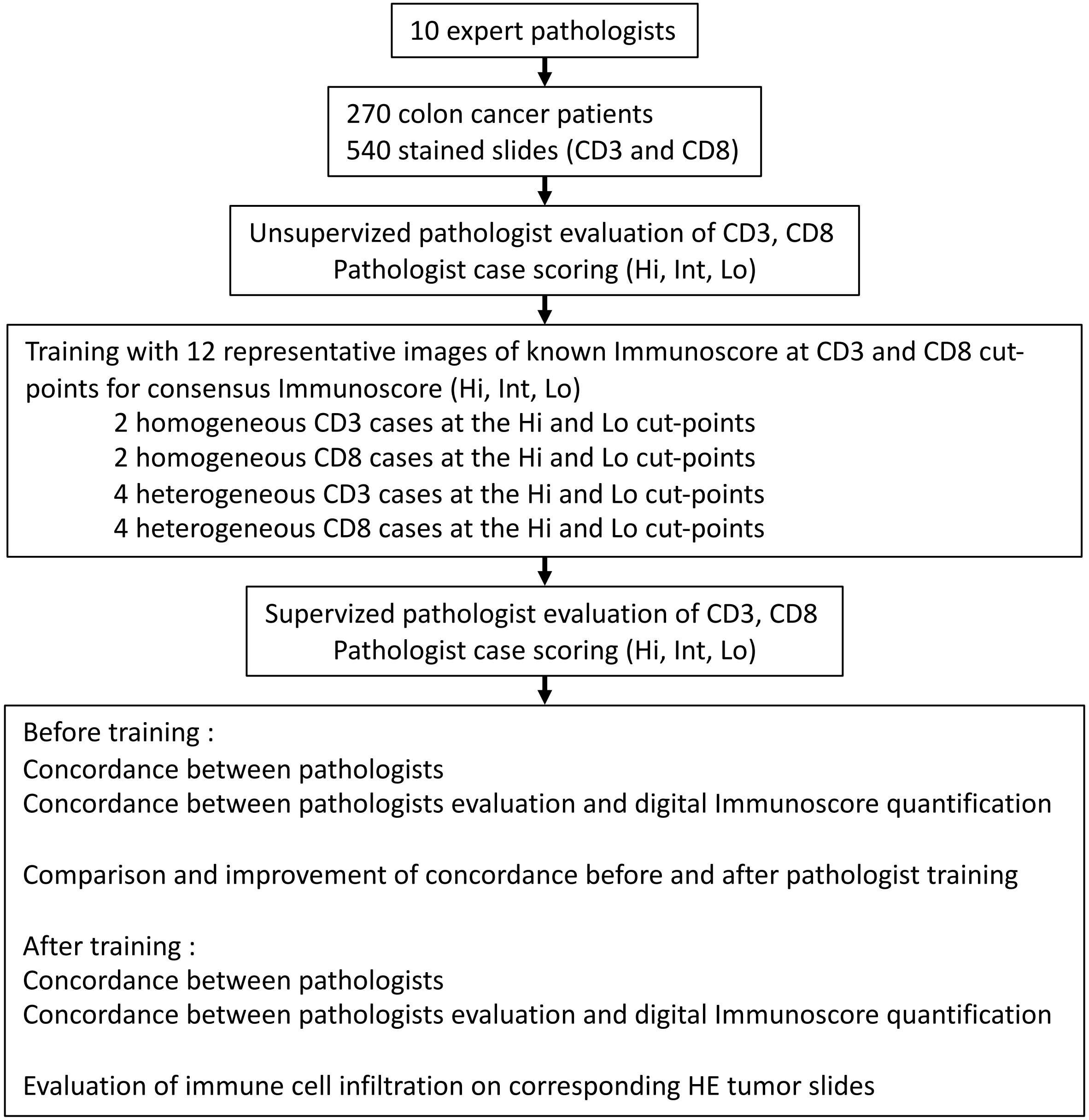

Supplementary Figure S1

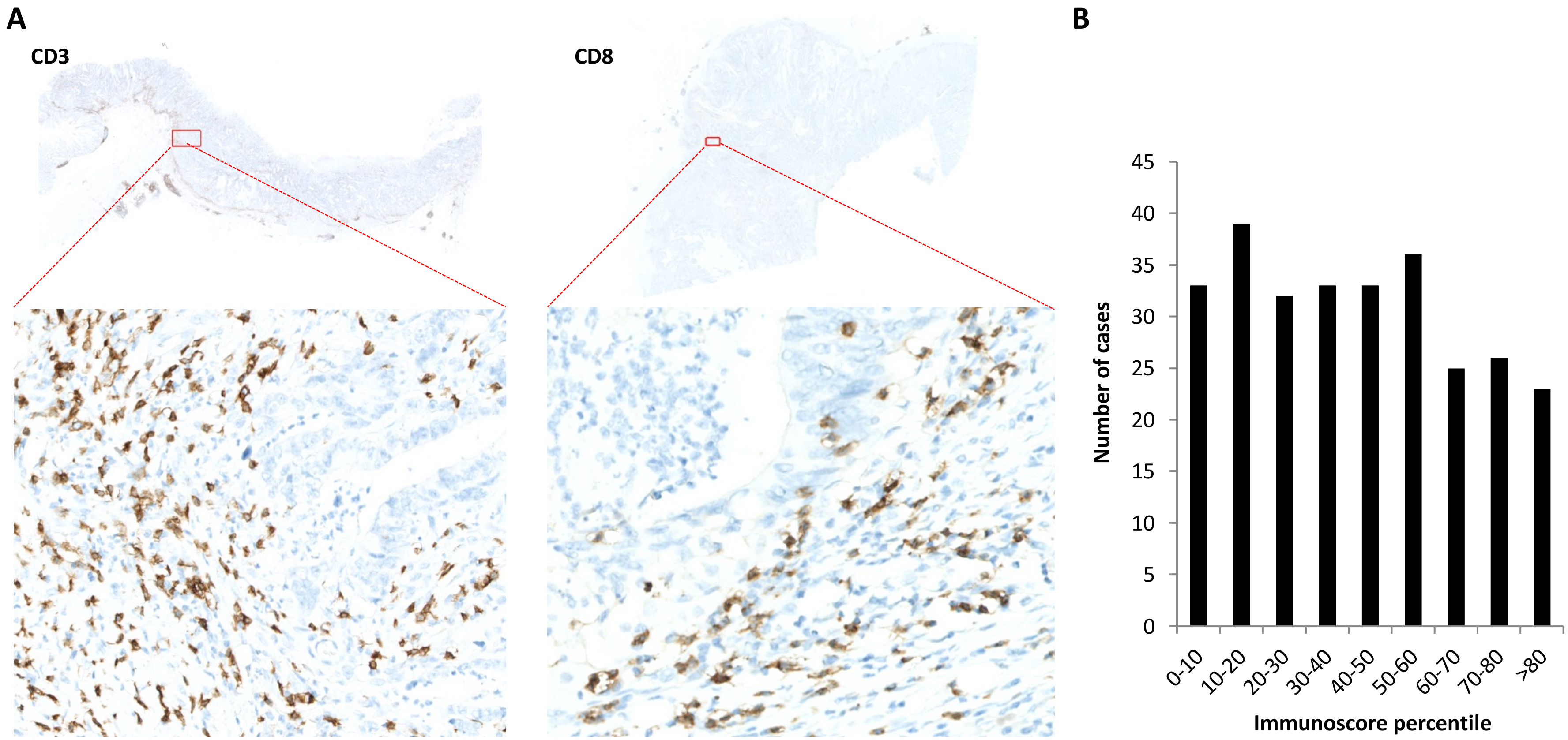

Supplementary Figure S2

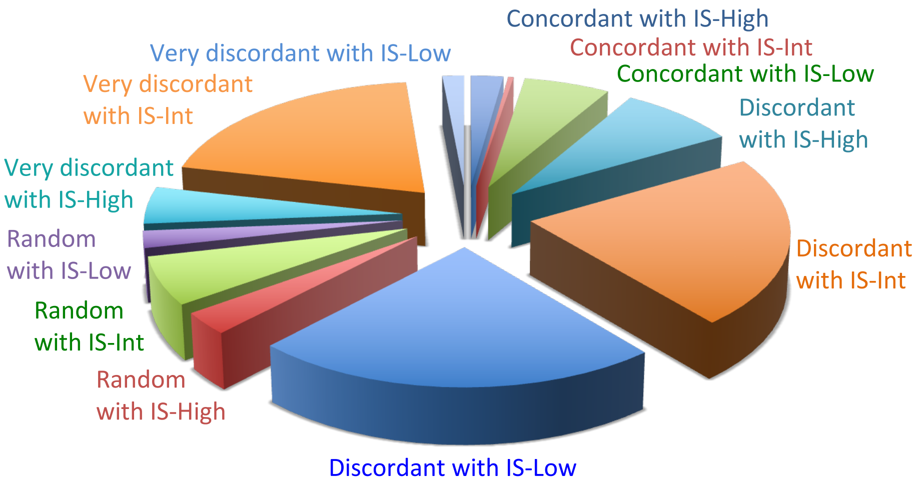

Supplementary Figure S3

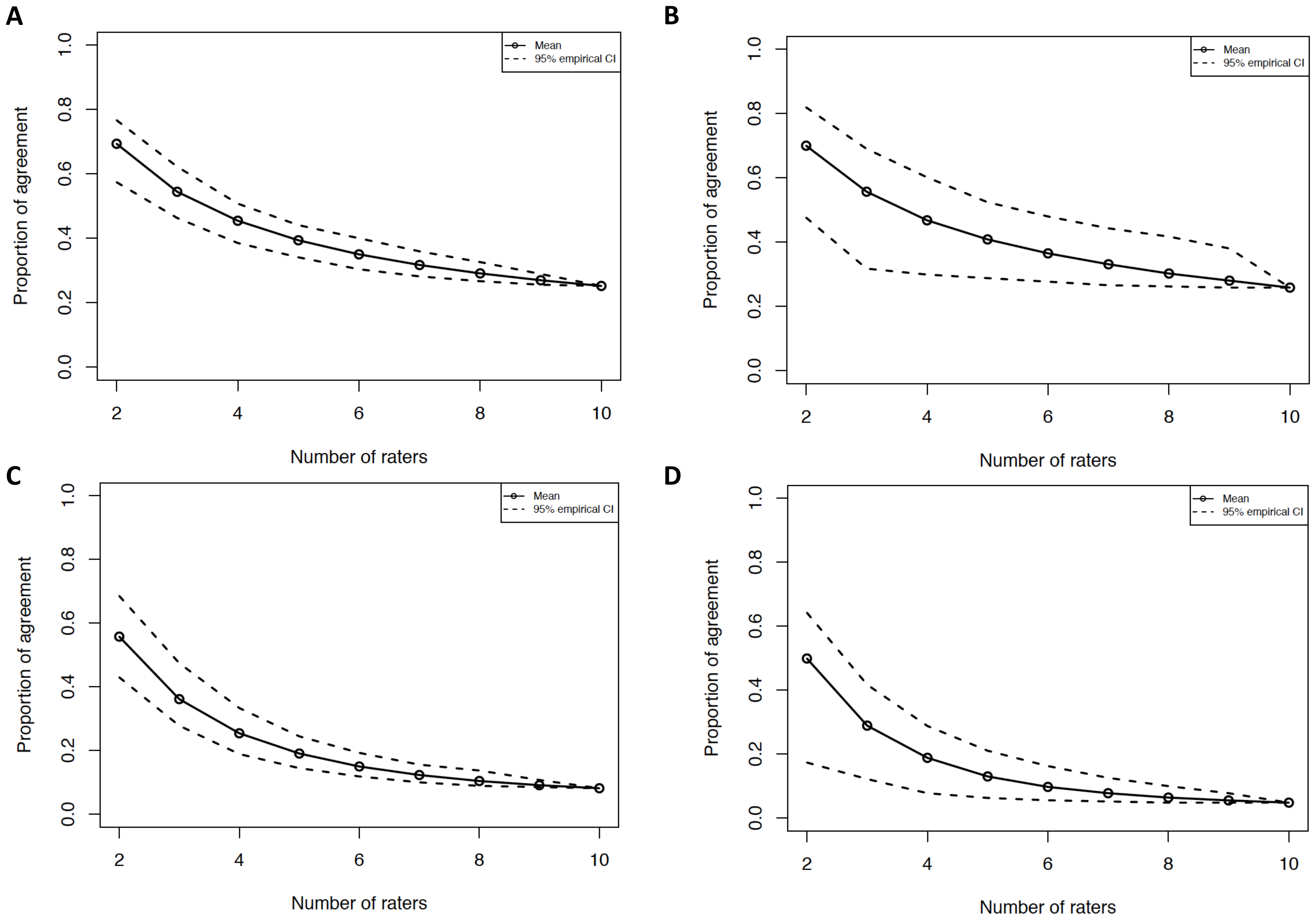

Supplementary Figure S4

Patient 1:
T4 N0 M0
0 Positive Lymph-node out of 10
MSS
Recurrence, Death

One typical IS-High patient where pathologist visual-scoring were not concordant

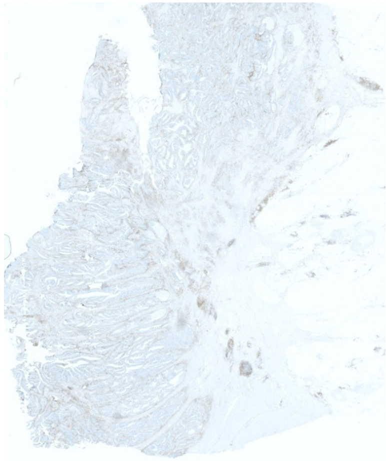

Immunoscore (Digital)
IS Category: High
IS: 71.25%

Pathologist Visual T-score

#1: Int
#2: Int
#3: Int
#4: High
#5: Int
#6: Int
#7: Int
#8: High
#9: High
#10: Low

Supp Figure S5A

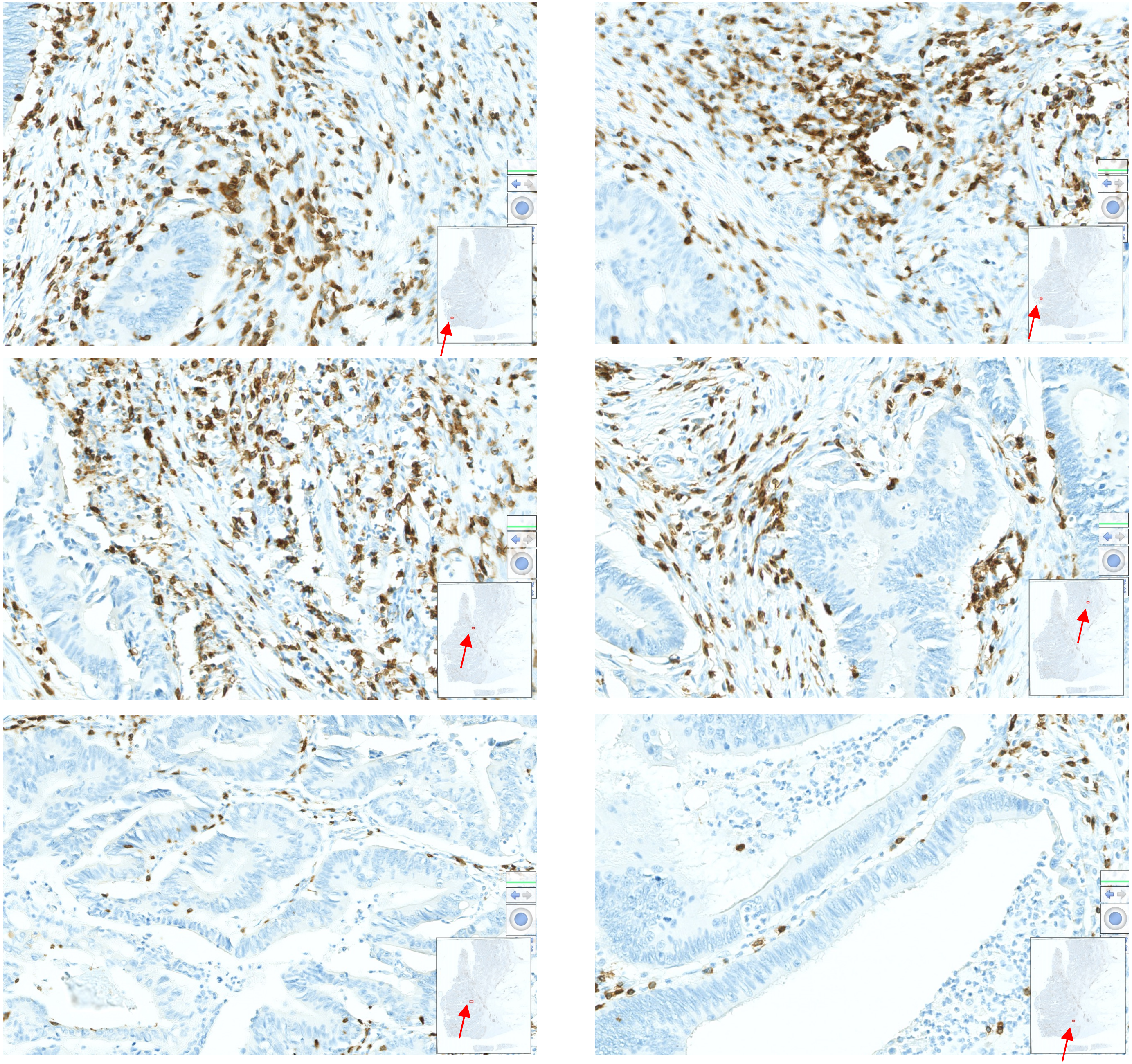

Patient 2:
T2 N0 M0
0 Positive Lymph-node out of 17
MSS
No recurrence, no death

One typical IS-Int patient where pathologist visual-scoring were not concordant

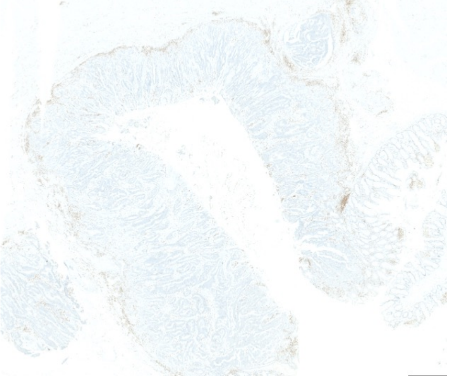

Immunoscore (Digital)
IS Category: Int
IS: 42.25%

Pathologist Visual T-score

#1: High
#2: Int
#3: Int
#4: Int
#5: Low
#6: Int
#7: Low
#8: High
#9: Low
#10: Int

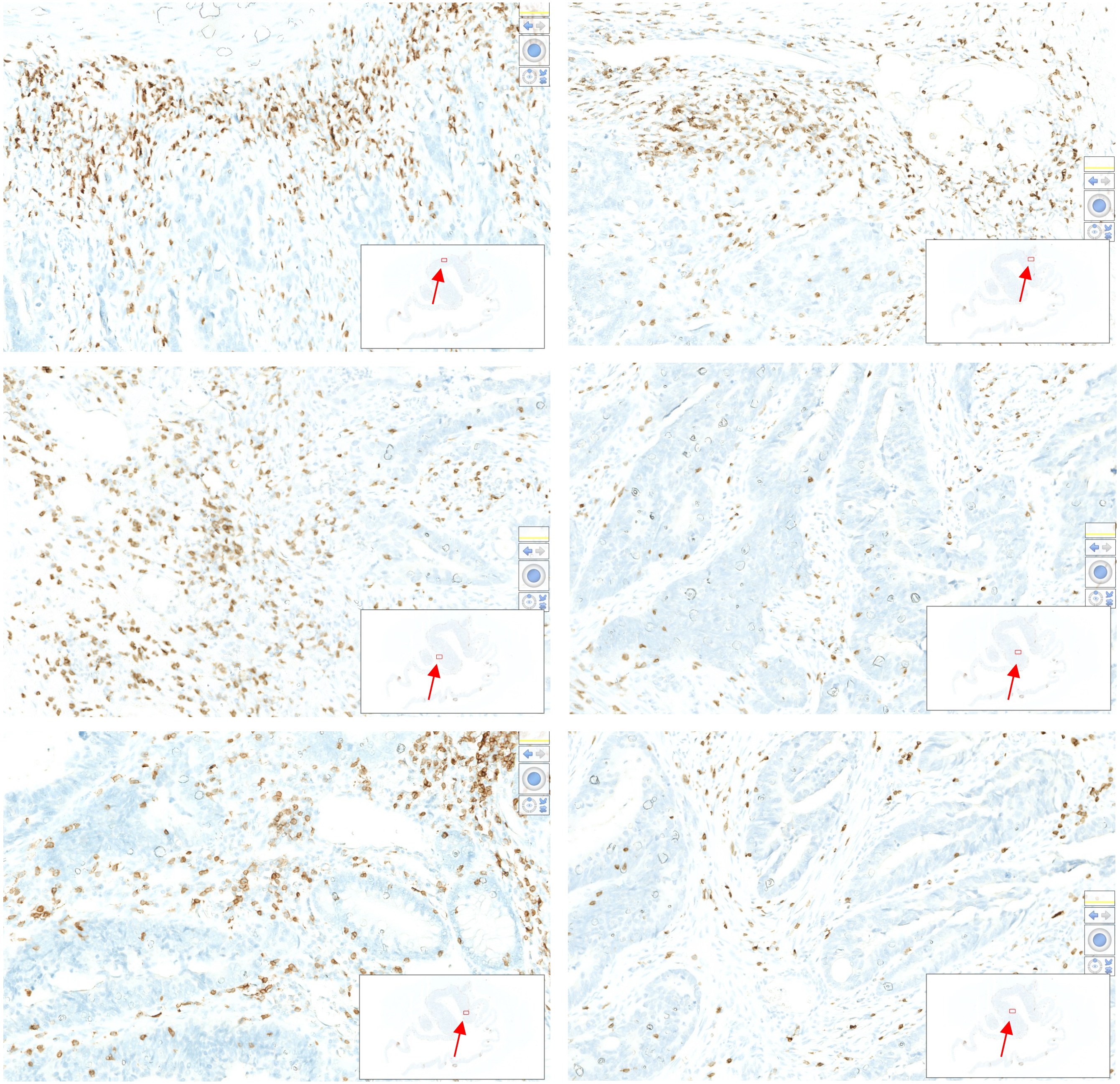

Supp Figure S5B

Patient 3:
T4 N0 M0
0 Positive Lymph-node out of 15
MSI-H
No recurrence, no death

One typical IS-Int patient where pathologist visual-scoring were not concordant

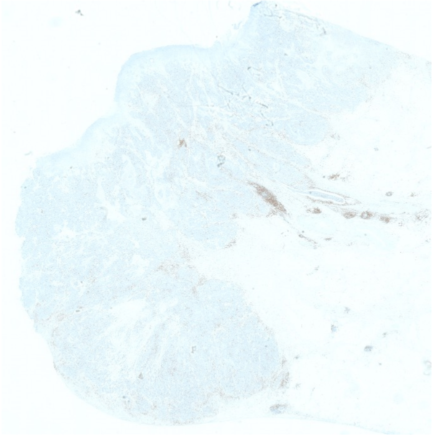

Immunoscore (Digital)
IS Category: Int
IS: 63.75%

Pathologist Visual T-score

#1: Int
#2: Low
#3: Int
#4: High
#5: Int
#6: Int
#7: Low
#8: High
#9: High
#10: Low

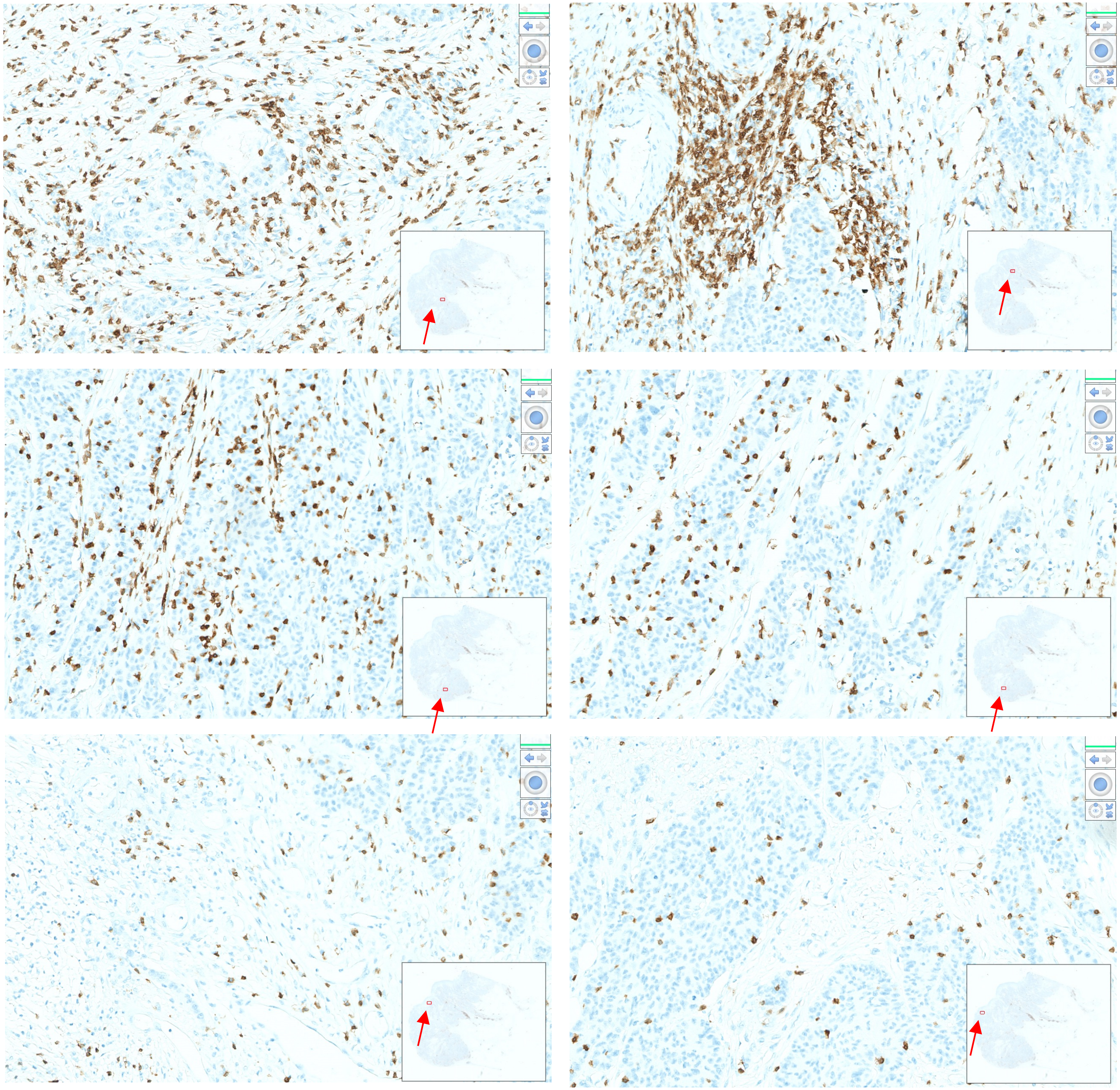

Supp Figure S5C

Patient 4:
T4 N0 M0
0 Positive Lymph-node out of 10
MSS
Recurrence, Death

One of the few extreme IS-Low patient where all pathologist visual-scoring were concordant

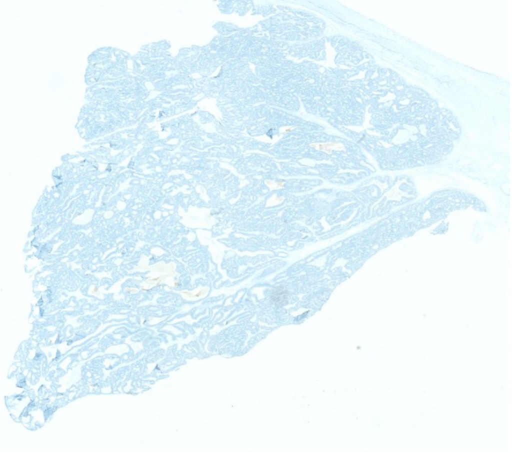

Immunoscore (Digital)
IS Category: Low
IS: 2.5%

Pathologist Visual T-score

#1: Low
#2: Low
#3: Low
#4: Low
#5: Low
#6: Low
#7: Low
#8: Low
#9: Low
#10: Low

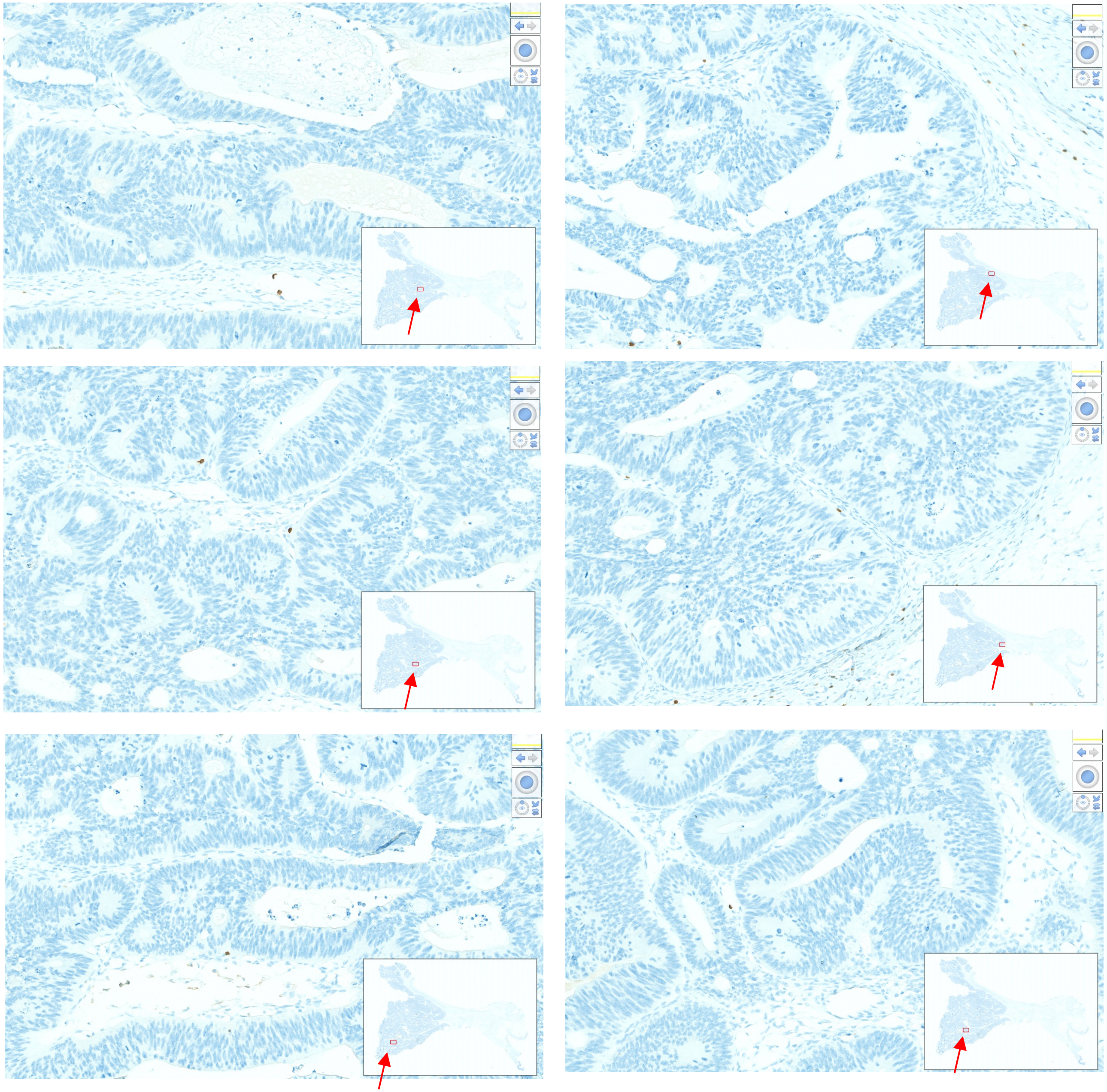

Supp Figure S5D

Patient 5:
T3 N2 M0
15 Positive Lymph-node out of 25
MSS
No recurrence, no death

One of the few extreme IS-High patient where all pathologist visual-scoring were concordant

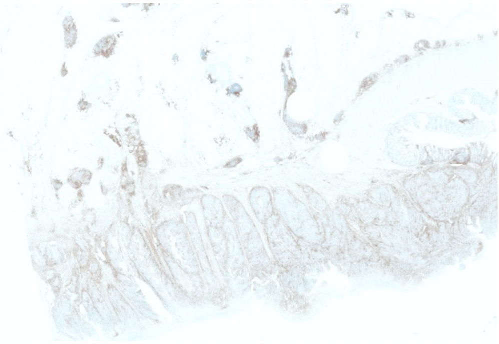

Immunoscore (Digital)
IS Category: High
IS: 97.5%

**Pathologist**	**Visual T-score**
#1:	High
#2:	High
#3:	High
#4:	High
#5:	High
#6:	High
#7:	High
#8:	High
#9:	High
#10:	High

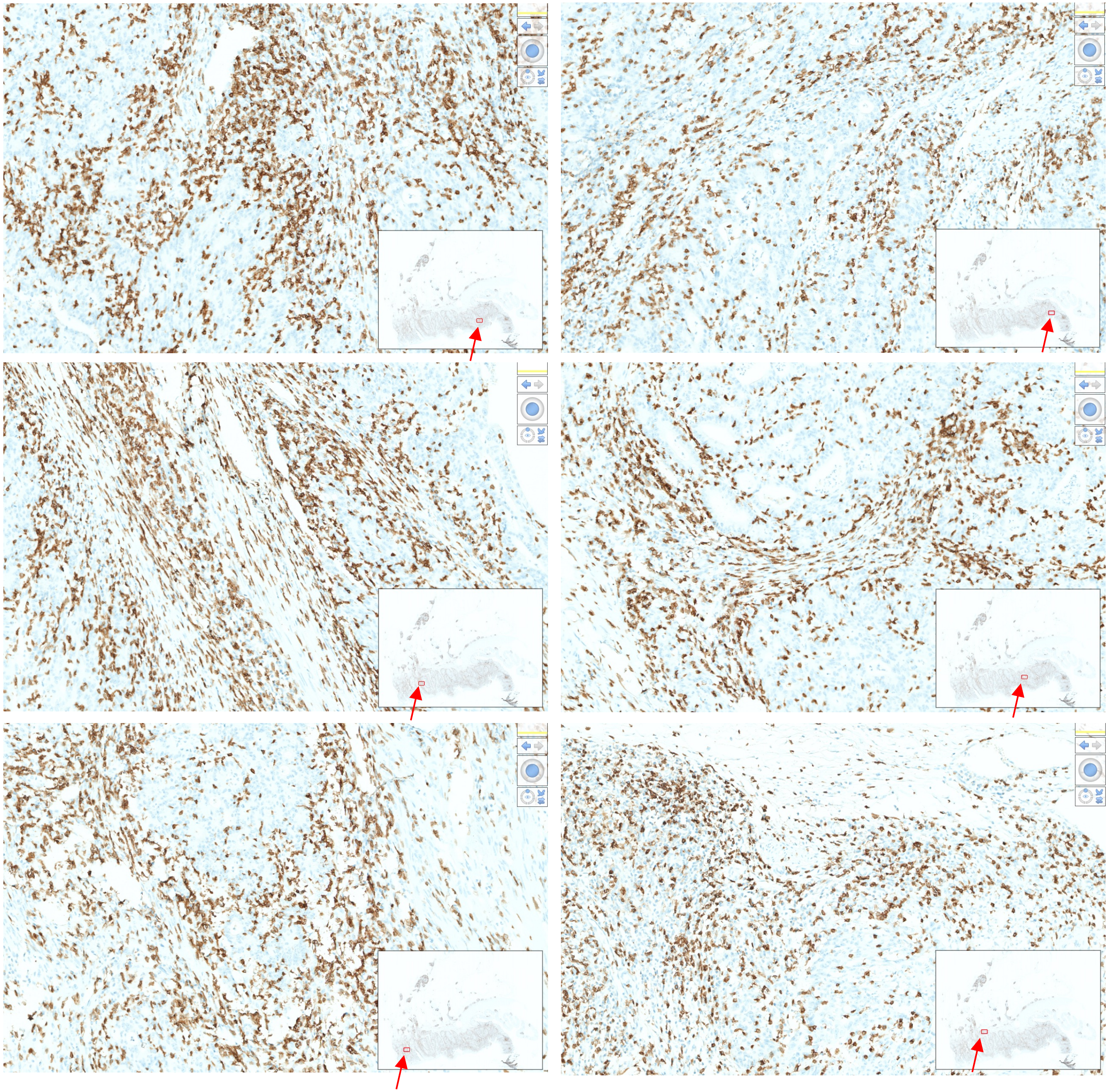

Supp Figure S5E

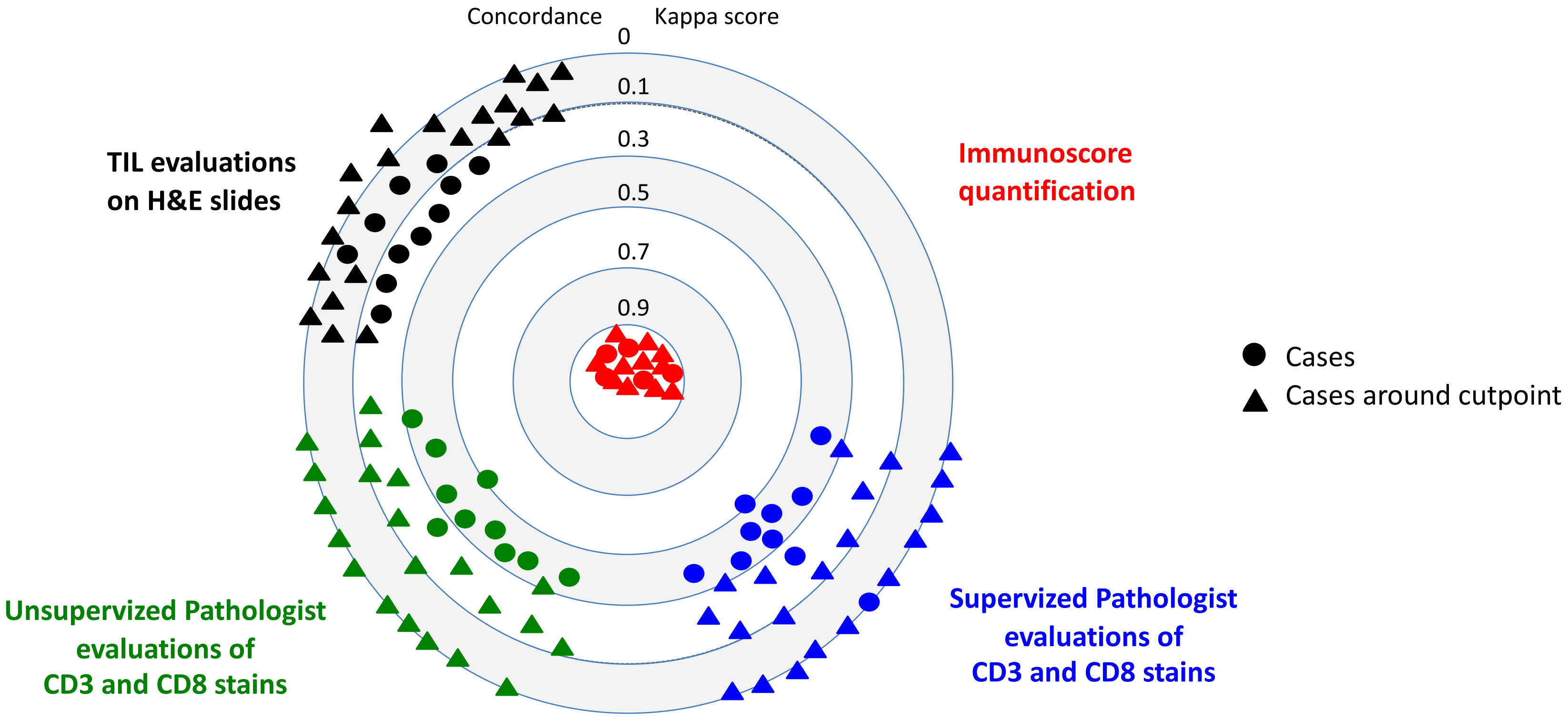

Supplementary Figure S6

A

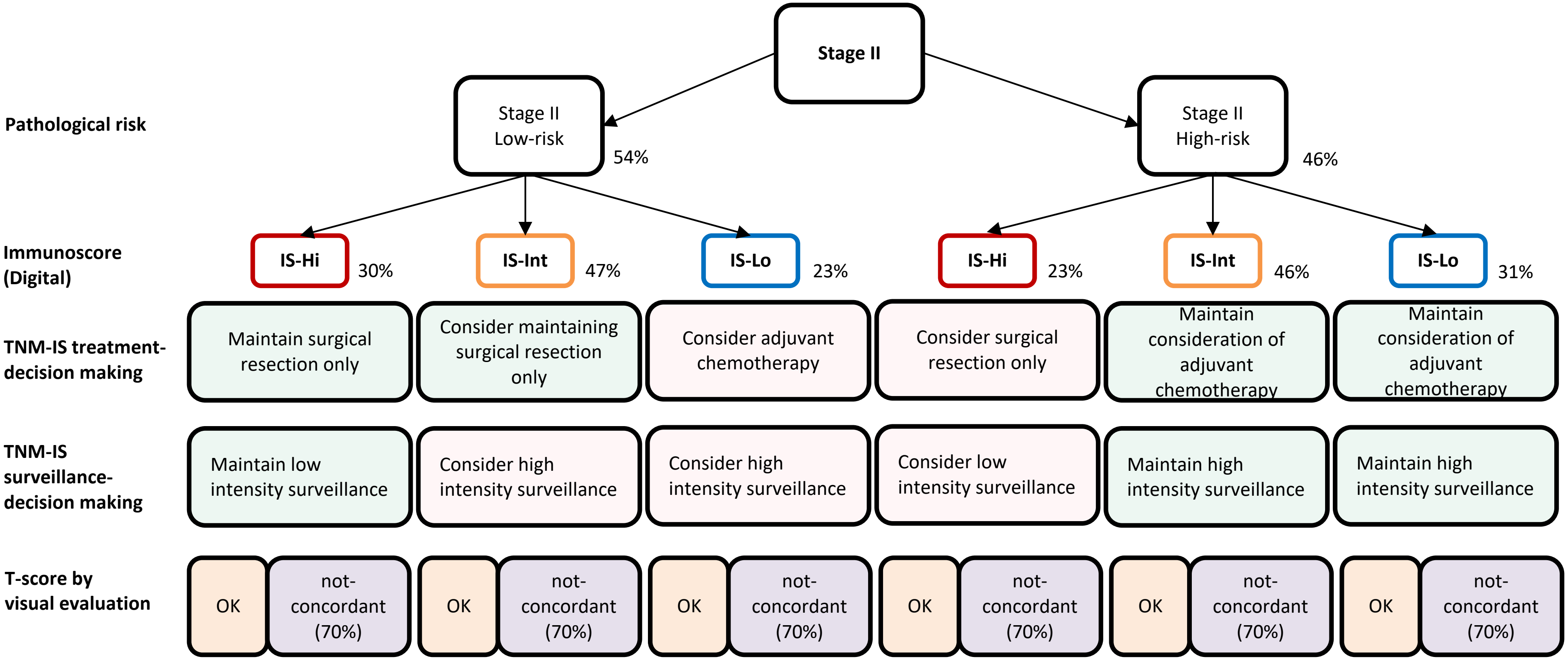

- Immunoscore (IS) could impact treatment decision-making between 23-48% of the patients with stage II colon cancer
- Immunoscore (IS) could impact surveillance decision-making for 48% of the patients with stage II colon cancer
- Visual evaluation of T-score by pathologist would lead to 70% of cases not concordant leading to inapropriate treatment and surveillance

Supplementary Figure S7A

B

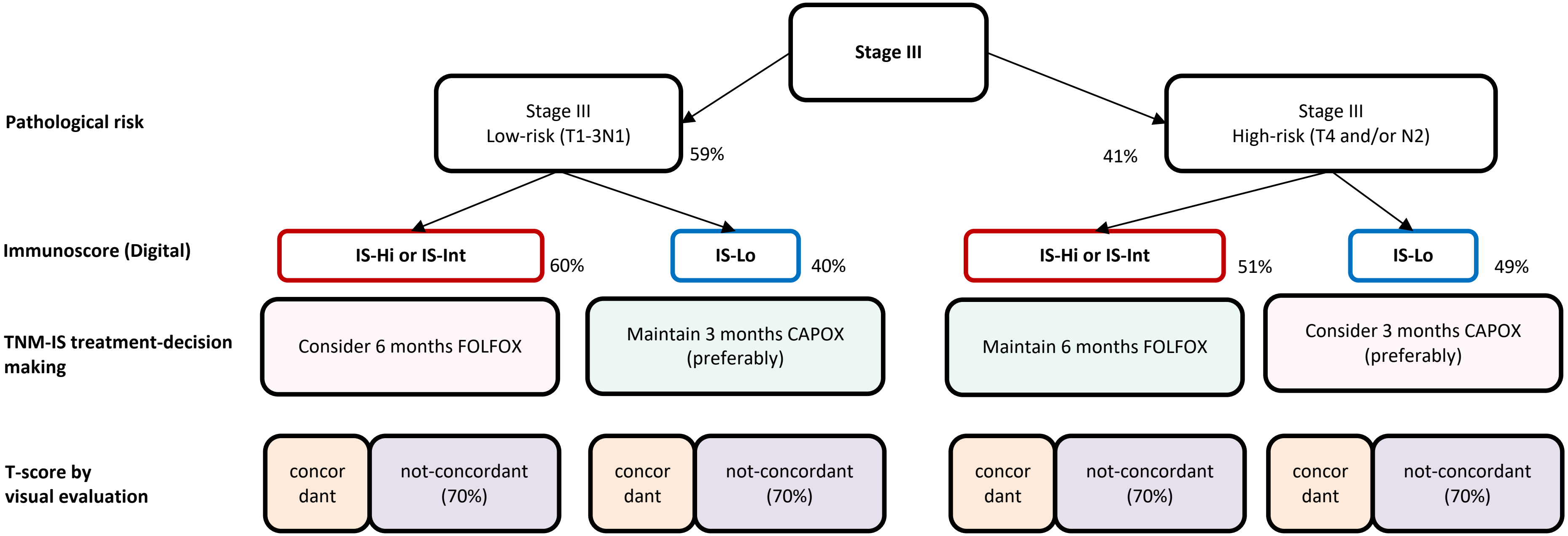

- Immunoscore (IS) could impact treatment decision-making for 55% of the patients with stage III colon cancer
- Visual evaluation of T-score by pathologist would lead to 70% of cases not concordant leading to inapropriate treatment and surveillance

Supplementary Figure S7B

Supplementary Table S1. Cohort characteristics and patients' demographic distribution.

Supplementary Table S2. Cohen's Kappa statistical analysis highlighting agreements between pathologists' T-score and the reference IS for clinical subgroups of colon cancer patients (*see Table 1 for details*).

Supplementary Figure S1. Detailed schematic representation of the experimental design.

Supplementary Figure S2. Representative images of CD3 and CD8 staining. (A) Paraffin sections of the colon tumor and invasive margin from each patient were processed by immunohistochemistry, and the densities of CD3+ and cytotoxic CD8+ T cells in the tumor and in the invasive margin were quantified by pathologists (T-score) or digital pathology (IS). (B) For each case, CD3 and CD8 densities in CT and IM regions were converted into percentiles.

Supplementary Figure S3. Detailed concordance analysis between individual pathologist's T-score. Pie chart representing concordance levels for every IS category (Low, Intermediate, High). For a less fragmented view, *cf.* Figure 1B, 1C.

Supplementary Figure S4. ONEST plot showing T-score overall percent agreement between pathologists as a function of the number of observers. Mean percent agreement ± 95% SD from all possible combinations are illustrated, using two category cutoffs (A, B), three category cutoffs (C, D), with unsupervised (A, C) or supervised analyses after training (B, D).

Supplementary Figure S5. Representative images of CD3 staining from 5 cases: non-concordant patients (5A, 5B, 5C), concordant patient with Low-IS (5D) and concordant patient with High-IS (5E). For each patient, pathologists' visual T-score, IS category, and patient clinical data (T, N, M, MSI, number of lymph-node, recurrence and death) are indicated. For each case, 6 different high-magnification zones of the same tumor illustrate CD3 staining. The red arrow in each panel points to the specific region of the whole tumor zoomed in. CD3 staining is shown highly heterogenous in the different tumor regions of non-concordant patients 1, 2 and 3 (5A, 5B, 5C). Similar observations are obtained with CD8 staining for different patients' cases.

Supplementary Figure S6. Target plot summarizing the study. Cohen's Kappa scores illustrating agreements between pathologists' T-score and the reference IS (3 categories) for 270 colon cancer patients, before and after supervised training. ** Kappa: worse than random (negative Kappa scores), none (0-0.2), weak (0.4-0.59), moderate (0.6-0.79), strong (0.8-0.9) and almost perfect (>0.9). Each dot illustrates one observer. Each triangle illustrates evaluation of cases around the cut-points by one observer, as previously described in Figures 2 and 3.

Supplementary Figure S7. Treatment and surveillance clinical decision-tree according to IS in Stage II (7A) and Stage III (7B) patients.
